# The Aggregate Point Rule for Identifying Shifts on P Charts and U Charts

**DOI:** 10.1097/pq9.0000000000000103

**Published:** 2018-09-20

**Authors:** T. Arthur Wheeler, J. Terrance Davis, Richard J. Brilli

**Affiliations:** From the * Quality Improvement Department, Nationwide Children’s Hospital, Columbus, Ohio; † Nationwide Children’s Hospital, Columbus, OH; ‡ Department of Surgery, The Ohio State University College of Medicine, Columbus, Ohio; § Division of Pediatric Critical Care Medicine, Department of Pediatrics, Nationwide Children’s Hospital, The Ohio State University College of Medicine, Columbus, Ohio.

## INTRODUCTION

Many valuable tools currently used in health care quality improvement (QI) have been borrowed from industry, initially from nuclear power and commercial aviation, where high reliability is essential.^1–4^ QI data require statistical analysis to determine if interventions have caused a system change that improved performance. A common statistical approach to accomplish that involves a concept borrowed from manufacturing: statistical process control (SPC).^5–8^ Data are gathered regarding specific processes and the points are plotted on an SPC chart, also known as a control chart, a process-behavior chart, or a Shewhart Chart—after its inventor, Shewhart.^9^ In a large majority of cases, more than 99% of observations of a stable process will fall within 3 SDs of the mean, although the exact percentage is variable for the types of charts addressed in this article, dependent upon actual process mean and sample sizes. Thus, any points outside of those limits suggest a “special cause” indicating that the process at that point in time is not stable—something has changed significantly,^10^ which, depending on the process, could be good or bad.

Visual rules have been described for viewing SPC charts that help detect significant change simply by viewing the chart.^10–13^ A total of 10 rules have been described (Table 1).^5^ All these rules identify points, or combinations of points, that represent special cause. In QI work, a relevant question is: when have enough of these points occurred over a sufficient duration to establish a new centerline representing sustained change? That is referred to as a centerline shift. Although a single point outside of the 3 SD control limits is considered special cause, it would not be sufficient evidence, in itself, of a centerline shift. For example, it might simply have been because of a key individual going on a brief vacation. Establishment of a centerline shift is generally more than a mere mathematical exercise and requires evaluation of the overall pattern of the data points and knowledge of the process.

**Table 1.**
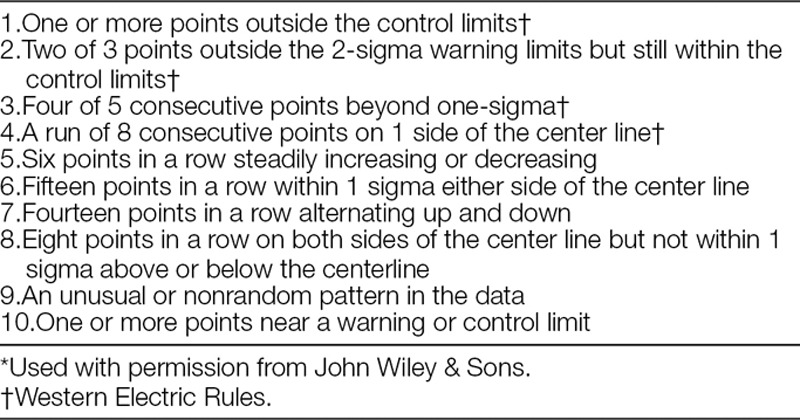
Rules for Identification of Special Cause Variation in Shewhart Control Charts^5^*

Many centerline shifts can be identified by applying 1 or more of the 10 rules cited above. Some QI experts may be facile with all 10 of the visual rules, and some SPC software has the ability to identify data points on a control chart affected by these rules. However, that knowledge is not common among the majority of individuals working on pediatric QI projects. In fact, it has been our experience that over the years, the “eight-point rule” (EPR)^10,13^ (sometimes 7 or 9, depending on the set of rules being used) has become the predominant way to identify a centerline shift—that is, the process has shifted if 8 or more consecutive points occur on the same side of the center line. This practice has evolved despite the fact that it has not been recommended in any SPC literature. The EPR is a probability-based rule, roughly corresponding to *P* < 0.01. That means: using standard cumulative binomial probability calculations, the probability that random variation alone would yield 8 consecutive points on the same side of the center line is less than 1 in 100, leading to the conclusion that a process shift has occurred. The EPR is easy to use, visualize, and remember. It is depended upon not only by QI novices but by experienced QI experts. As an example of the prevalence of EPR usage, the “Solutions for Patient Safety” national collaborative of 130 pediatric hospitals has used the EPR to identify centerline shifts since its beginning, and still does today (personal communication). Although the EPR has served well, it has several limitations when used exclusively to evaluate QI projects in the health care environment.

- The time required to satisfy the EPR can be long. In health care, QI data are frequently reported or aggregated monthly or quarterly. Unlike manufacturing, where processes can be completed in minutes or hours, many measured processes in health care occur less frequently (eg, clinic visits, surgical procedures). When data are recorded monthly, it takes at least 8 months to amass 8 consecutive points above or below the center line (2 years if recorded quarterly). Unlike quality control in manufacturing, where the goal is to document stability, or detect change should it occur, QI tries to create change, so early identification of even a small center line shift is highly valuable feedback. The time necessary to satisfy the EPR is a down side to that technique.
- The EPR can miss many smaller shifts entirely if they don’t yield 8 consecutive points on the same side of the center line. For example, consider 7 points above, followed by 2 below, then followed by 6 points above a center line. Using cumulative binomial probability,^5^ one can demonstrate that this sequence (13/15 points above the center line) has an even (slightly) lower probability of occurring by chance than 8 consecutive points above the centerline. Yet it would not be recognized by the EPR.
- The EPR assumes approximately equal probability of a point falling above or below the center line. Violations of this assumption are not serious in many circumstances, but can be extreme on p charts and u Charts if rates are close to zero (or close to 100% on p charts). For example, consider an Adverse Drug Event rate with a centerline of 0.295 per 10,000 doses and a daily volume of 6,600 administered doses. Cumulative binomial probability calculations demonstrate that it would require 25 consecutive daily points below the center line to satisfy *P* < 0.01 for a downward shift if plotted as a daily chart with a denominator of 6,600, whereas the same data plotted on a monthly chart with ~200,000 doses per point requires only 7 consecutive points.
- The EPR doesn’t take into account how far points are from the center line, yet the distance from the centerline enters into the calculation for the probability that those points could have occurred by chance in an in-control process: as a group of points, the farther from the center line, the less likely the process is still stable. For example, 6 points more than 2 sigma away from the center line represent special cause. If the points are monthly, it would be difficult not to regard them as a legitimate shift (as opposed to transient fluctuation).

Although some of these limitations may be avoided by applying one or more of the other visual rules, the EPR tends to be the primary rule explicitly associated with identifying shifts.^11^ The obvious danger in using this rule exclusively, is that, while the conditions for satisfying the EPR are usually sufficient to confirm a valid process shift, they are not always necessary and, in fact, can be unduly restrictive.

Another simple, easy-to-use rule, as an alternative or complement to the EPR, could be very useful in identifying a wider range of process shift situations. And while “the infusion of new ideas into the accepted body of SPC knowledge has been very slow,” in part because of “the comfort of existing systems in professional quality circles,”^14,15^ we have been using an alternative statistical test for p charts and u charts. This test is simple, easy to perform, and can detect centerline shifts before 8 consecutive data points occur, or in other situations where the EPR is never satisfied. We call this test the Aggregate Point Rule (APR). This test applies a 3-sigma criterion—essentially a “control limit”—for the set of data points being tested, rather than for single points. This group “control limit” identifies whether that dataset constitutes a special cause as supported by a 3-sigma criterion,^16^ suggesting a possible process change, assuming other criteria delineated below are met.

## APR

**Fig. 1.**
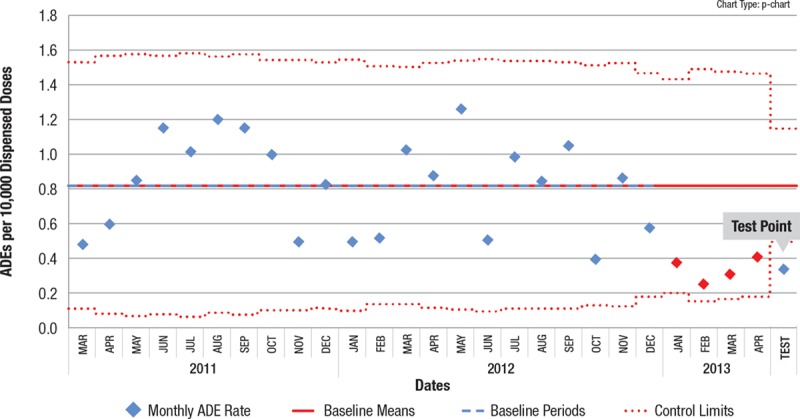
Control chart with APR test point. The test point outside the control limit confirms special cause indication for the red points, and thus potentially a process shift, depending on the process and the calendar time involved for the points.

**Fig. 2.**
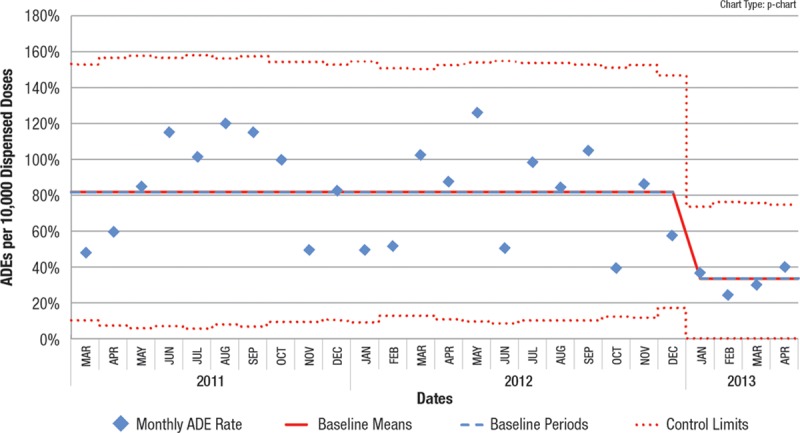
Test point is removed and new centerline indicated.

1. Using standard software to generate a p chart or u chart (QI Macros Add-in for Excel, by KnowWare International, Inc. <www.qimacros.com> or similar software), ensure that a stable baseline has been established.
2. Identify the set of points (“dataset”) to test that seem to potentially represent a change from that baseline. For example, consider a group of points near 2 sigma from the centerline—depicted as the red data points in Fig. 1. For practical reasons, do not consider fewer than 4 points on the same side of the center line. If the points are not all on the same side of the center line you may need more than 8 points overall.
  Note: Points under consideration should not be points used in the center line calculation (blue data points in Fig. 1). If they are, they will degrade your ability to detect a shift because they will raise (or lower) the center line value you’re comparing against, thus underrating the magnitude of the shift. Furthermore, by its nature, a shift represents a new process level.
3. Define an aggregate point, designated the “test point”, that represents the test dataset (see the blue “test point” in Fig. 1:
  - Numerator of test point = Sum of individual point numerators for the dataset.
  - Denominator of test point = Sum of individual point denominators for the dataset.
4. Plot the test point on your control chart. You can plot the point immediately after the points that you are testing to see if a shift has occurred (“see Fig. 1). The appropriate limits for a p chart or u chart will be automatically calculated based on the combined denominator.
5. If this test point is outside its corresponding control limit, as in Fig. 1, then it represents special cause, just as any other point outside a control limit would. Subject matter experts can then evaluate overall patterns on the chart to decide if this special cause represents a shift.
6. If a shift is identified, remove the temporary point and start a new center line through the tested points as demonstrated in Fig. 2. The new centerline should be periodically updated until data are sufficient to constitute a stable centerline.

**Fig. 3.**
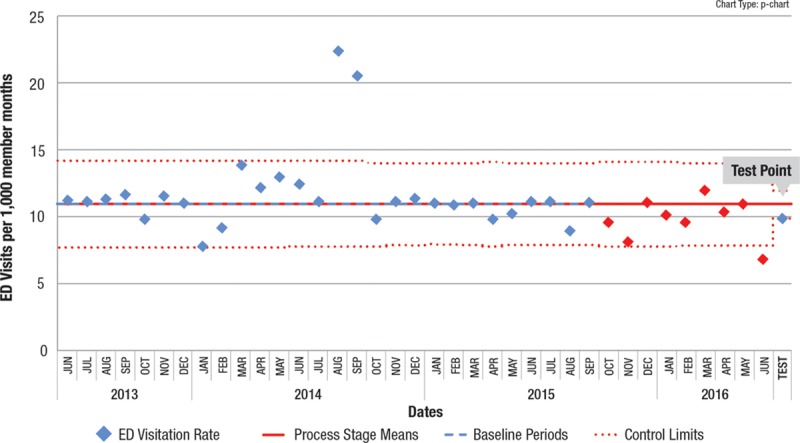
Control chart where the 9 points in red (beginning in October 2015) would not have satisfied the EPR because 8 consecutive points are not below the line, yet the test point of the APR shows special cause, suggesting a centerline shift.

**Fig. 4.**
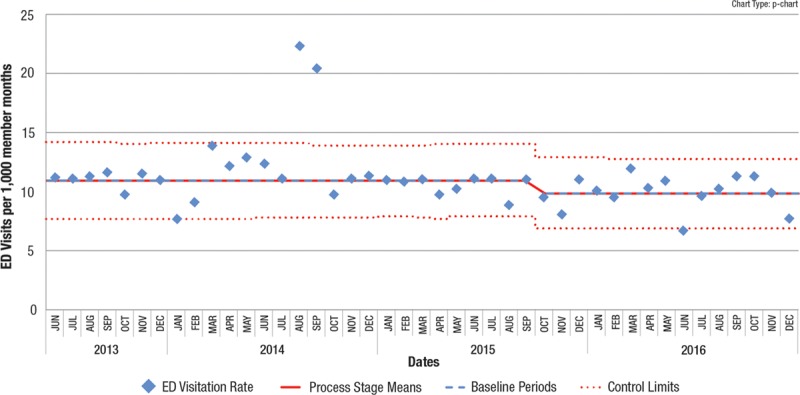
Subsequent control chart showing new centerline and an additional 6 months of data all part of the new level.

We suggest that using this APR technique in conjunction with the EPR will identify a higher percentage of valid process shifts than the EPR alone, and identify them earlier. In addition to identifying shifts before 8 points, the APR can identify a centerline shift when 8 consecutive points never occur, as exemplified by data from an actual recent QI project. In Fig. 3, beginning in October 2015, 8 consecutive points never occur below the centerline, but performing the APR on data points from October 2015 to June 2016 successfully identified special cause. Fig. 4 shows the resultant centerline shift and demonstrates that special cause continued to be recognized after June 2016 as each successive point was added. In this situation, adhering to the EPR would not have identified the centerline shift, even 15 months after the shift was identified by the APR. Earlier (and enhanced) process shift detection allows the QI process to accelerate, speeding up subsequent testing of other interventions, or enabling spreading the improved process across the organization.

Note that in both of these examples, other standard control chart rules would identify the possibility of a shift. In the first example, the “2 out of 3 points close to the control limits (outer 1/3 of chart)” is indicated (first 3 red points). In the second example, there is a point below the lower control limit. These examples illustrate the problem mentioned earlier of someone only using the EPR to identify a centerline shift.

## DISCUSSION

We have used the APR for over 10 years, and it has proved extremely valuable in identifying valid process shifts sooner than would result by rigidly adhering only to the EPR. Moreover, like the EPR, it provides a simple method of evaluating a potential centerline shift. The only expertise required is the creation of the aggregate test point from the data points being tested. The software automatically calculates the control limits to evaluate the aggregate point.

The APR, unlike other rules, does not specify a precise number of points or exactly when to apply the rule. As noted above, that is determined by the user after examining the pattern of data points in the initial chart. Given that determination, the APR makes use of standard statistical relationships to determine whether a temporary “aggregate” data point is within 3 SDs of the mean. Because control limits on a p chart or u Chart are sigma based (plus or minus 3 SDs),^16^ the narrower limits on the test point reflect its larger denominator.

Although the APR technique involves an element of subjectivity to properly administer, that subjectivity lies not in whether you have special cause and a potential centerline shift, but rather in deciding whether the special cause is of sufficient duration to constitute a sustained centerline shift. This is true in all good control chart practice, not just with the APR, and in our QI experience this has not been problematic.

The APR is not intended to replace the EPR or any of the other previously described rules. It is simply proposed as an alternate statistical approach that offers the advantages of earlier detection of many process shifts, detection of otherwise undetected smaller shifts, and simplicity. It does not require the use of any additional statistical tools or different types of charts. Nor does it require plotting the SPC chart in zones or sigma levels. This approach is, in some ways, similar to alternative (but less familiar) charting methods—for example rational subgrouping (eg, going to quarterly versus monthly subgroups to get tighter limits). Other methods such as Cumulative Sum or Exponential Weighted Moving Average charts^6^ are typically recommended for detecting smaller shifts. However, the APR does not require learning and understanding a new type of chart. Therefore, this is an easily understood alternative because virtually all QI team members understand basic control charts and process shifts.

## CONCLUSIONS

An alternative technique, the APR, is described as a simple, additional way to detect center line shifts in QI data. It is not as restrictive as the EPR, and can identify shifts in as few as 4 or 5 points. It can also identify smaller shifts in longer sequences when 8 consecutive points do not fall on the same side of the center line. It is easy to perform and understand using standard control chart methodology. Earlier and more comprehensive shift detection resulting from this process has made the APR an integral part of interpreting our QI control charts.

## DISCLOSURE

The authors have no financial interest to declare in relation to the content of this article.
